# Insomnia and Other Disorders of Sleep

**Published:** 1885-11

**Authors:** 


**Insomnia and other Disorders of Sleep.** By HENRY M. LYMAN, A. M., M. D., Professor of Physiology and Diseases of the Nervous System in Rush Medical College, Professor of Theory and Practice of Medicine in the Woman's Medical College, and Physician to the Presbyterian Hospital, Chicago, Ill. Chicago: W. T. Keener. 1885.

This work, of seven chapters, on the nature and cause of sleep, insomnia, remedies for insomnia, treatment of insomnia in particular diseases, dreams, somnambulism, artificial somnambulism, or hypnotism, contains a resumé of these subjects; although, in our judgment, the book would be of greater value if it set before its readers more careful and detailed directions for the exhibition of certain remedies in disease. The subject of insomnia is one that is little studied, and for which physicians prescribe in the most routine manner. A work such as this, with the addition of carefully prepared and attested experiments, such as would give the practitioners some guide for prescribing this or that remedy in the various diseases, would be of great value to the profession. As it is, we think it will be useful to those who have not given the subject much thought, as an introductory work.

